# The genetics of divergence and reproductive isolation between ecotypes of *Panicum hallii*

**DOI:** 10.1111/nph.13027

**Published:** 2014-09-23

**Authors:** David B Lowry, Kyle Hernandez, Samuel H Taylor, Eli Meyer, Tierney L Logan, Kerrie W Barry, Jarrod A Chapman, Daniel S Rokhsar, Jeremy Schmutz, Thomas E Juenger

**Affiliations:** 1 Department of Integrative Biology and Institute for Cellular and Molecular Biology, University of Texas at Austin1 University Station C0990, Austin, TX, 78712, USA; 2 Division of Science and Environmental Policy, California State University, Monterey Bay100 Campus Center, Seaside, CA, 93955, USA; 3 Center for Research Informatics, University of Chicago, McGiffert House5751 S. Woodlawn Avenue, Chicago, IL, 60637, USA; 4 Biology Department, Bowdoin CollegeBrunswick, ME, 04011, USA; 5 Department of Zoology, Oregon State UniversityCordley Hall 3029, Corvallis, OR, 97331, USA; 6 Department of Energy, Joint Genome Institute2800 Mitchell Drive, Walnut Creek, CA, 94598, USA; 7 Center for Integrative Genomics and Department of Molecular and Cell Biology, University of California at BerkeleyBerkeley, CA, 94720, USA; 8 HudsonAlpha Genome Sequencing Center601 Genome Way, Huntsville, AL, 35806, USA; 9 Department of Plant Biology, Michigan State University, Plant Biology Laboratories612 Wilson Road, Room 166, East Lansing, MI, 48824, USA

**Keywords:** adaptation, drought, ecotype, physiology, pleiotropy, quantitative trait locus (QTL), reproductive isolation

## Abstract

- The process of plant speciation often involves the evolution of divergent ecotypes in response to differences in soil water availability between habitats. While the same set of traits is frequently associated with xeric/mesic ecotype divergence, it is unknown whether those traits evolve independently or if they evolve in tandem as a result of genetic colocalization either by pleiotropy or genetic linkage.
- The self-fertilizing C_4_ grass species *Panicum hallii* includes two major ecotypes found in xeric (var. *hallii*) or mesic (var. *filipes*) habitats. We constructed the first linkage map for *P. hallii* by genotyping a reduced representation genomic library of an F_2_ population derived from an intercross of var. *hallii* and *filipes*. We then evaluated the genetic architecture of divergence between these ecotypes through quantitative trait locus (QTL) mapping.
- Overall, we mapped QTLs for nine morphological traits that are involved in the divergence between the ecotypes. QTLs for five key ecotype-differentiating traits all colocalized to the same region of linkage group five. Leaf physiological traits were less divergent between ecotypes, but we still mapped five physiological QTLs. We also discovered a two-locus Dobzhansky–Muller hybrid incompatibility.
- Our study suggests that ecotype-differentiating traits may evolve in tandem as a result of genetic colocalization.

## Introduction

The process of speciation often occurs as a continuum over time through the accumulation of reproductive isolating barriers that prevent gene flow between new species (Dobzhansky, 1937; Clausen, 1951; Wu, 2001; Schemske, 2010; Nosil, 2012). A common intermediate stage in the process is the evolution of partially reproductively isolated ecotypes, which result from adaptation to different habitats (Turesson, 1922; Clausen, 1951; Grant, 1981; Lowry, 2012; Nosil, 2012). Reproductive isolation between ecotypes is often caused by ecologically based prezygotic isolation (Lowry *et al.*, 2008; Nosil, 2012). However, postzygotic isolation can also occur between ecotypes as a result of chromosomal rearrangements or epistatic Dobzhansky–Muller interactions (Coyne & Orr, 2004).

In plants, soil water availability plays a dominant role in the distribution and productivity of species worldwide (Stebbins, 1952; Whittaker, 1975; Woodward *et al.*, 2004). Thus, it is not surprising that ecotype formation in plants is frequently driven by divergent adaptations to habitats that differ in soil water availability (Clausen & Heisey, 1958; Porter, 1966; Lowry, 2012; Andrew *et al.*, 2013). Adaptation to soil water availability can drive the evolution of divergence in physiology, development, morphology, phenology, life-history, and reproductive allocation strategies between plant ecotypes (Stebbins, 1952; Clausen & Heisey, 1958; Baker, 1972; Roux *et al.*, 2006; Juenger, 2013). Ecotypes that are adapted to mesic habitats typically have leaves with greater area, more axillary branching, and are larger overall than ecotypes adapted to drier habitats (Clausen & Heisey, 1958; Kruckeberg, 1986; Rajakaruna, 2004; Lowry, 2012). These classic differences in morphology suggest a trade-off between above-ground growth, particularly of leaves, and the demand for water imposed by transpiration, which results from a greater transpiring surface area (Geber & Dawson, 1990; Dudley, 1996; Ackerly *et al.*, 2000). Ecotypes occurring in dry habitats frequently evolve to develop fast and flower early, a life history that avoids periods of drought (Ludlow, 1989; McKay *et al.*, 2003; Franks, 2011). Further, plants adapted to drier habitats typically have larger seeds than those adapted to wetter habitats, which reflects a trade-off between offspring provisioning and total reproductive output (Baker, 1972).

While there is often a common set of morphological and life-history traits that differentiate xeric and mesic ecotypes (Lowry, 2012) across plant species, very little information is known about the genetic basis of parallel evolution among ecotype-differentiating traits. One major unresolved question is whether the common set of traits involved in mesic vs xeric ecotype divergence evolves in tandem, as a result of colocalization of genetic effects to particular chromosomal regions, or whether these traits can evolve independently of each other. Recently, a study in the yellow monkeyflower, *Mimulus guttatus*, identified a major quantitative trait locus (QTL) that controlled the divergence in many traits differentiating xeric and mesic ecotypes (Hall *et al.*, 2006; Lowry & Willis, 2010). This major QTL mapped to a large chromosomal inversion, which suggests that genetic linkage may play a role in the concerted evolution of many ecotype-differentiating traits (Kirkpatrick, 2010). Similar colocalizing QTLs were also found in a cross between xeric and mesic accessions of *Arabidopsis thaliana* (McKay *et al.*, 2003, 2008; Lovell *et al.*, 2013). One of these colocalizing QTLs appears to be the result of a mutation in *FRIGIDA*, and suggests that pleiotropy through mutations in major regulatory genes can be responsible for the divergence of multiple traits related to drought adaptations (Lovell *et al.*, 2013). QTL colocalization has also been reported for traits involved in divergence between xeric and mesic ecotypes of *Avena barbata* (Latta & Gardner, 2009). Beyond these few examples, it is unknown how often colocalization of QTLs occurs for the common set of traits involved in xeric vs mesic ecotype divergence.

Here, we examine whether there is genetic colocalization of traits involved in the divergence of morphological, phenological, leaf physiological, and reproductive allocation traits between two ecotypes of the C_4_ perennial grass, *Panicum hallii* Vasey. We also evaluated potential mechanisms of reproductive isolation that maintain these ecotypes despite their overlapping geographic ranges. *P. hallii* is a highly self-fertilizing (Mean *F*_*IS*_ = 0.895; Lowry *et al.*, 2013) rangeland species that occurs across much of the southwestern USA and northern Mexico. There is a considerable population genetic structure among populations of *P. hallii* and among major biogeographical regions within its geographical range (Lowry *et al.*, 2012, 2013). However, the greatest morphological divergence within *P. hallii* occurs between its two ecotypes, which have been previously classified by taxonomists as distinct varieties (Gould, 1975; Waller, 1976)*. P. hallii* var. *hallii* (Scribn.) Waller is the most widespread ecotype and is typically located in water-limited (xeric) upland calcareous habitats (Gould, 1975; Waller, 1976). By contrast, *P. hallii* var. *filipes* (Scribn.) Waller is primarily found in mesic depressions and is more geographically restricted, most often found in the Rio Grande Valley and along the Gulf Coast Plain of Texas and Mexico (Gould, 1975; Waller, 1976; Hatch *et al.*, 2003). The two ecotypes co-occur in areas where both wet and dry microsites are available (Waller, 1976), but are morphologically distinct (Lowry *et al.*, 2013), especially in south Texas (Waller, 1976). Consistent with differential adaptation to wet and dry environments (Baker, 1972; Dudley, 1996; Ackerly *et al.*, 2000; Picotte *et al.*, 2007; Lowry *et al.*, 2008; Maherali *et al.*, 2009), var. *hallii* has smaller leaves, smaller overall plant size, earlier flowering time, and larger seeds than var. *filipes* (Waller, 1976; Lowry *et al.*, 2013).

The primary aim of our study was to understand the genetic basis of multi-trait divergence and reproductive isolation between var. *hallii* and var. *filipes* through QTL mapping. To conduct QTL mapping in this system, we developed a linkage map using a new restriction site-associated DNA (RAD) genotyping method that utilizes IIB restriction enzymes (Wang *et al.*, 2012). This map also allowed us to also evaluate the extent of chromosomal synteny between *P. hallii* and foxtail millet (*Setaria italica*), which is the closest relative of *P. hallii* with a published sequenced genome (Zhang *et al.*, 2011; Bennetzen *et al.*, 2012). Overall, we addressed the following four major questions: what genomic regions contribute to the morphological and leaf physiological trait variation/divergence within and among var. *hallii* and var. *filipes*? Do QTLs for multiple ecotype-differentiating traits cluster (colocalize) in particular chromosomal regions? What are the patterns of synteny between *P. hallii* and *S. italica* and is there evidence for colocalization of flowering time QTLs between those two species? What reproductive isolating barriers might account for maintenance of divergence between var. *hallii* and var. *filipes*?

## Materials and Methods

### Development of the F_2_ FIL2 ×  HAL2 mapping population

To better understand the genetic basis of divergence between *Panicum hallii* var. *hallii* and var. *filipes*, we created an F_2_ mapping population. The parents of the mapping populations were FIL2 and HAL2-11. FIL2 is a genotype derived from a natural var. *filipes* collection made near the Corpus Christi Botanical Gardens in South Texas (27.65°N, 97.40°W), and is the primary line being sequenced by the Joint Genome Institute (DOE) for var. *filipes* (Meyer *et al.*, 2012). HAL2-11 is a one-generation selfed progeny of HAL2, which was derived from a natural collection made at the Lady Bird Johnson Wildflower Center (Austin, TX, USA; 30.19°N, 97.87°W), and is the primary line being sequenced for var. *hallii*. All of the F_2_ plants used in this study were derived as self-fertilized progeny from a single F_1_ hybrid, where FIL2 was the sire and HAL2-11 was the dam in the cross (see Supporting Information Methods S1 for details of the crossing methodology). We refer to HAL2-11 as HAL2 throughout the remainder of the manuscript because this lineage is highly homozygous and, thus, quite similar across generations.

### Morphological phenotyping

We scarified F_2_ seeds with sandpaper and placed them on white paper towels moistened with water in Petri dishes sealed with parafilm on 18 July 2011. To synchronize germination the Petri dishes were placed in a refrigerator at 4°C, to stratify seeds. On 25 July 2011 the Petri dishes were moved to a growth chamber for germination using the same methods as Lowry *et al*. (2013). As seeds germinated they were transferred to a 60 : 40 mixture of Promix (Premier Tech Horticulture, Rivière-du-Loup, Québec, Canada): Turface (Profile Products, Buffalo Grove, IL, USA) in 4 inch square pots. The first seedlings were transplanted on 3 August 2011, and the last on 23 August 2011. Plants were then grown under 16 h days maintained by supplemental fluorescent lighting in the University of Texas glasshouses. Seedlings were arrayed in a fully randomized design and trays were rearranged weekly to minimize the effects of environmental heterogeneity across the experiment.

We measured four traits on the first flowering tiller at anthesis: tiller height, tiller thickness, flag leaf length, and flag leaf width. A flag leaf is the upper most leaf on a tiller and subtends the inflorescence. We also counted the number of tillers at anthesis. Flowering time was calculated as the date of transplantation subtracted from the date of first flower. Once the inflorescence of the first flowering tiller had fully expanded we measured the length of the inflorescence (first branch point to tip) and counted the number of flowers on the inflorescence. We calculated seed mass as the mean weight of 10 viable seeds collected from each F_2_ plant. We observed that many of the F_2_ plants had low levels of seed production or produced no apparent viable seed, and we scored sterility as a binary trait, with 1 indicating at minimum dozens of viable looking seeds (i.e. hard, dark colored seed coat) and a 0 indicating fewer than five viable looking seeds (i.e. soft, light colored seed coat).

### Physiological phenotyping

We measured leaf physiological traits on 214 unique F_2_ lines, 18 clonally propagated FIL2, and 19 clonally propagated HAL2 individuals (see Methods S1 for details of environmental conditions). We aimed to determine efficiencies with respect to photosynthesis and leaf water use; higher photosynthetic capacity can help plants to maximize photosynthesis in dry environments, and differences in efficiency can be linked with leaf lifespan through variation in nutrient and structural investments (Wright *et al.*, 2003; Osnas *et al.*, 2013).

Survey measurements were made with two LI-6400XT portable photosynthesis systems (Li-Cor Inc., Lincoln, NE, USA) at ambient CO_2_ (reference CO_2_ controlled at 400 μmol mol^−1^; sample CO_2_, 386 ± 6 μmol mol^−1^ (mean ± SD)) to obtain values for net CO_2_ assimilation (*A*)_,_ stomatal conductance (*g*_*s*_), and leaf intercellular CO_2_ concentrations (*c*_*i*_). Maximization of *A* relative to *g*_*s*_ will reduce *c*_*i*_, and all else being equal the capacity to maintain lower *c*_*i*_ indicates a greater ratio of carbon fixation to water lost for a leaf (Farquhar & Richards, 1984). Quantum efficiency of photosystem II (Φ_PSII_) as well as its factors: photochemical quenching (q_P_) and the light adapted efficiency of energy harvesting by open PSII reaction centers (*F*_v_′/*F*_m_′), were measured concurrently with gas exchange using fluorometers integrated into the cuvette lids (LI-6400-40). These fluorescence parameters are indicators of the operating efficiency of PSII (Maxwell & Johnson, 2000), thus linear electron transport in the light reactions of photosynthesis; when incident light is similar, higher values for fluorescence parameters indicate that a leaf is making more effective use of absorbed light and, therefore, its investment in light harvesting capacity.

During gas exchange, a chlorophyll SPAD meter (Konica-Minolta SPAD 502; Konica-Minolta, Chiyoda, Tokyo, Japan) was used to measure each leaf adjacent to the section being measured for gas exchange, and a mean value was recorded. SPAD values are a dimensionless index of leaf chlorophyll content determined from the ratio of transmitted light with wavelengths 940 and 650 nm (Markwell *et al.*, 1995), that is, a coarse indication of the investment in light harvesting capacity.

Before mapping of physiological traits, we independently excluded outliers for gas exchange, fluorescence, and SPAD among the 214 F_2_ individuals. When measuring gas exchange, estimates of *g*_*s*_ in particular can be influenced by a variety of sources of measurement error, thereby influencing estimates of water-use efficiency. We therefore eliminated measurements of gas exchange that indicated substantial deviation from the expected relationships between *A* and *g*_*s*_. The top 5% (11) values for *c*_*i*_ were removed; these values were >254 μmol mol^−1^, which is outside of the normal range for C_4_ photosynthesis (we would expect *c*. 150 μmol mol^−1^). We further removed gas exchange data representing the bottom 5% of residuals (7) from a regression of *A* on *g*_*s*_ that included random effects of the machines used on both the slope and intercept. Fluorescence traits for one plant were eliminated from the dataset due to an unusually low *F*_v_′/*F*_m_′ (gas exchange data for this plant had already been eliminated due to unusually high *c*_*i*_). Seven SPAD values falling outside a 99% confidence interval around the mean (SPAD values 17.5–42.5) were removed. The same filters were applied to the FIL2 and HAL2 data, and gas exchange values for one FIL2 parent with *c*_*i*_ > 254 μmol mol^−1^ were removed.

Subsequent to outlier exclusion, best linear unbiased predictors (BLUPs) for each F_2_ were generated for physiological traits, using mixed-effects models (lme4, Bates *et al.*, 2013) to correct for the estimated random effects of the machine used and the day on which each measurement was taken.

### Genotyping and analysis of RAD markers

Following the quantification of morphological traits, we collected tissue from all F_2_ plants and extracted high-quality DNA using a DNeasy Plant Mini Kit (Qiagen, Valencia, CA, USA). To obtain markers for mapping, we prepared our DNA with a recently developed 2b restriction site–associated DNA (2b–RAD) genotyping method (Wang *et al.*, 2012). This method utilizes type IIB restriction enzymes, which cut both upstream and downstream of the enzyme's target site and results in the production of RAD tags of uniform length. We used the *Alf*I restriction enzyme for preparation of our RAD libraries. We followed the reduction protocol of Wang *et al*. (2012) to target only 1/16th the *Alf*I sites across the genome for sequencing based on modified adaptors. We sequenced the RAD tags on both the Illumina and SOLiD (Applied Biosystems, Foster City, CA, USA) platforms in pools of barcoded individuals (range: 39–105 individuals per pool).

To create a reference genome for mapping of RAD tags, we conducted genome sequencing of the FIL2 *P. hallii* var. *filipes* accession with the Illumina GAIIx and Illumina HiSeq platforms (Illumina Inc., San Diego, CA, USA). Genomic DNA was extracted at the University of Texas at Austin and sequenced at the Department of Energy Joint Genome Institute (JGI) in Walnut Creek, CA, USA. DNA was prepared for sequencing with insert sizes ranging from 0.2 to 35 kbp and paired-end 76, 100, and 150 bp reads (Table S1). Preliminary scaffolds were assembled with Meraculous (Chapman *et al.*, 2011), the JGI plant assembler, as part of a genome sequence project for *P. hallii* (used with permission). Raw sequence reads used in the Meraculous assembly can be found in the NIH sequence read archive (SRA) under project number SRP003932.

Trimmed and filtered reads (custom scripts: https://github.com/kmhernan/Publications) were mapped to the Meraculous assembly with SHRiMP mapping software (v2.2.3; David *et al.*, 2011; global alignments with five significant hits per read) and pre-processed with the Picard software tools (http://picard.sourceforge.net/). Picard is a suite of utilities that are used to manipulate Sequence Alignment/Map (SAM) formatted files. To improve computational efficiency, we reduced the Meraculous assembly to include only scaffolds with at least five reads mapped across 25% of the population. We then applied the Genome Analysis Toolkit (GATK; McKenna *et al.*, 2010) to conduct variant discovery, genotyping, and filtering at a large scale. We used GATK indel realignment, base quality recalibration (after an initial run to estimate high-quality SNPs), and performed genotyping across all samples simultaneously using standard filtering parameters (DePristo *et al.*, 2011). We created an initial set of potential markers by filtering the variants resulting from GATK based on the following criteria: we converted individual genotypes with Phred-scaled genotype quality (GQ) of < 20 to Ns (i.e. missing data); we only retained sites with bi-allelic polymorphism across all individuals; we only retained sites that had been genotyped in both parents and were homozygous for different alleles in each parent (HAL2 vs FIL2); we eliminated sites with >75% missing data and sites that had segregation distortion of *P *<* *0.00005 in a chi-squared test. Following these filtering steps, 803 potential markers remained for linkage map construction. See Fig. S1 for a flowchart of the genotyping pipeline.

### Linkage map assembly and synteny analysis

We inferred linkage groups based on marker recombination fractions of 0.15, 0.1 and 0.05 using JoinMap 4 (Van Ooijen, 2006). We used the JoinMap regression mapping algorithm for linkage map construction with the following settings: pairwise recombination frequency < 0.4, LOD > 3, goodness of fit threshold = 5, ripple run after each marker placement, and the Kosambi mapping function to calculate genetic distances. Following this first round of linkage map construction, we removed markers that substantially affected the goodness of fit of the data (e.g. mean chi-squared contribution > 3). We purged markers to eliminate incorrect markers orders that we identified through visualization of recombination fractions with R/qtl (Broman *et al.*, 2003).

We explored synteny with the foxtail millet genome (*S. italica* v9.0) and *P. hallii* linkage map to verify marker orders. We used the relative position of markers in the two genomes to identify the location of large-scale chromosomal rearrangements. See Fig. S2 for a flowchart of the synteny analysis pipeline. Briefly, we compared the position of foxtail millet exons with the position of homologous exons in the reduced *P. hallii* Meraculous genome assembly (tblastx–threshold 999). To identify most closely related homologous exons, we filtered out the ‘best’ BLAST hits. We then searched for the positions in the reduced Meraculous *P. hallii* assembly where a SNP fell within a region and had significant BLAST results to a foxtail millet exon. We extracted a 200-bp window around these SNPs and used BLAST to reciprocally compare them with the set of foxtail millet exons (tblastx–threshold 999). Finally, we extracted reciprocal ‘best’ hits from both BLAST outputs to identify syntentic positions in the two genomes.

### QTL analyses

We conducted QTL mapping of morphological and physiological traits by stepwise fitting of multiple-QTL models in R/qtl (custom scripts: https://github.com/davidbryantlowry/panicumhalliiqtlmapping). Before model fitting, we calculated penalties for main effects and interactions for each trait through 1000 permutations of the scantwo function using Haley–Knott regression. We then used the stepwiseqtl function to conduct a forward/backward search using Haley–Knott regression for models with a maximum of six QTLs that optimized the penalized LOD score criterion. In addition to additive QTLs, the stepwiseqtl function considers models with all possible pairwise epistatic interactions. We calculated the 1.5–LOD drop interval of QTLs in the best-fit stepwise models using the lodint function. We also used the best-fit stepwise model for each trait to calculate the additive effect, dominance deviation, and percent of variance explained for each QTL with the makeqtl and fitqtl functions of R/qtl. Planting cohort was used as a covariate for QTL mapping of flowering time.

## Results

### Phenotypic variation and divergence

Consistent with previous studies (Waller, 1976; Lowry *et al.*, 2013), there was considerable divergence in traits between var. *hallii* (HAL2) and var. *filipes* (FIL2). For example, FIL2 plants had on average 1.8-fold longer tillers, 1.6-fold longer leaves, and took twice as long to flower as HAL2 plants (Table 1). While FIL2 plants had 4.5-fold more flowers, their seeds were only 40% of the size of the seeds of the HAL2 plants. There were significant correlations across the F_2_ population for 26 out of 36 pairwise comparisons of morphological traits (Table 2). We also found evidence for a hybrid incompatibility between the varieties, as 45 out of 229 (19.7%) F_2_ had a high level of sterility or were completely sterile.

**Table 1:** Means (SE) morphological trait values for FIL2, HAL2 and F_2_ *Panicum hallii* plants

Trait	*N*	FIL2	*N*	HAL2	*t*	*N*	F_2_	F_2_ range
Tiller height (cm)	12	65.70 (2.21)	30	35.61 (1.07)	−13.79****	246	46.88 (0.53)	28.6–80.0
Number of tillers	12	19.08 (1.41)	30	15.20 (0.68)	−2.78**	246	15.40 (0.34)	4–30
Tiller width (mm)	12	2.39 (0.08)	30	1.70 (0.04)	−8.43****	246	1.86 (0.02)	1.1–2.8
Leaf length (cm)	12	25.93 (1.86)	30	15.97 (0.30)	−8.00****	245	20.28 (0.28)	8.9–36.2
Leaf width (mm)	12	8.38 (0.39)	30	5.45 (0.12)	−9.52****	246	6.01 (0.07)	2.8–10.0
Flowering time (d)	12	85.08 (5.41)	30	43.53 (1.02)	−11.14****	246	60.85 (0.95)	36–105
Inflorescence length (cm)	9	24.02 (1.09)	30	21.19 (0.36)	−3.22**	243	22.27 (0.89)	14.5–35.6
Flower number	9	527.67 (51.02)	30	116.43 (6.63)	−13.85****	240	239.27 (5.94)	72–533
Seed mass (mg)	8	0.58 (0.01)	30	1.40 (0.01)	34.83****	200	0.91 (0.01)	0.65–1.24

*N,* number of replicates per parental lines or total number of F_2_ individuals (1 replicate per F_2_); *t*, *t*-statistic in test for divergence between parental lines

** *P* < 0.01

**** *P *< 0.0001.

**Table 2:** Significant pairwise morphological trait correlations in the FIL2 ×  HAL2 F_2_ population of *Panicum hallii*

	Tiller height	Tiller width	Tiller number	Leaf length	Leaf width	Flowering time	Inflorescence length	Flower number
Tiller height								
Tiller width	**0.356**							
Tiller number	**0.372**	**0.245**						
Leaf length	**0.308**	**0.511**	**0.280**					
Leaf width	**0.423**	**0.618**	**0.458**	**0.790**				
Flowering time	0.053	−0.016	−**0.467**	−**0.233**	−**0.297**			
Inflorescence length	**0.359**	**0.411**	**0.289**	**0.423**	**0.501**	−0.169		
Flower number	**0.472**	**0.510**	**0.440**	**0.478**	**0.624**	−**0.250**	**0.524**	
Seed mass	−0.027	0.065	0.125	0.096	0.145	−**0.349**	0.210	0.051

Significant pairwise Pearson product–moment correlations after Bonferroni correction at *P *<* *0.05 are indicated in bold.

**Fig 1:**
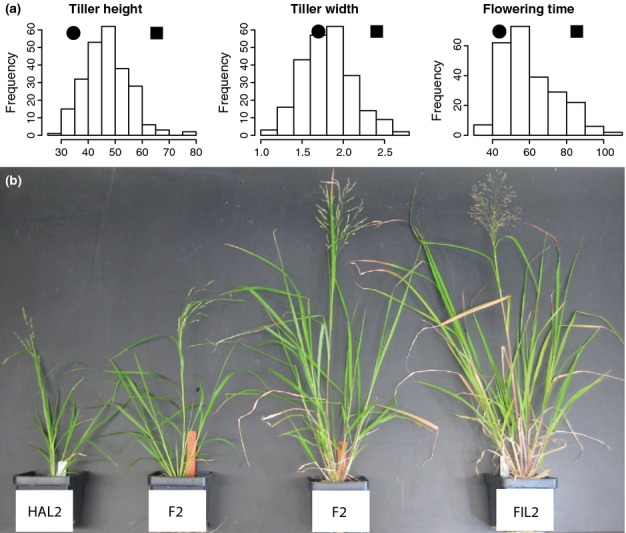
(a) The distribution of trait values in the F_2_ hybrid mapping population for tiller height, tiller width, and flowering time. The parental HAL2 (black circle) and FIL2 (black square) means are plotted above each distribution. (b) The divergence between *Panicum hallii* var. *hallii* (HAL2) and var. *filipes* (FIL2) was largely recovered in F_2_ individuals.

The distribution of morphological trait values in the F_2_ hybrids was generally bell-shaped, with a few traits, such as flowering time, having an extended tail in one direction (Fig. S3). The parental HAL2 and FIL2 means generally flanked the peak of the distribution of trait values (Figs 1a, S3). However, there was transgressive segregation for traits such as number of tillers and inflorescence length, where parental means were not as divergent (Fig. S3). Many of the extreme F_2_s in the distribution looked phenotypically similar to the parental phenotypes (Fig.1b).

Most physiological traits were not significantly different between FIL2 and HAL2 (Table 3), and there were high levels of transgressive segregation in the F_2_ hybrids (Table 3; Fig. S4). Of the photosynthetic traits measured, only *ci* showed a significant difference between the parental lines, being lower in FIL2 than HAL2 (Table 3). This implied that although differences in *A* (slightly greater in FIL2, Table 3) and *g*_*s*_ (smaller in FIL2, Table 3) were not significant, intrinsic water-use efficiency (*A* relative to *g*_*s*_) was greater in FIL2 than HAL2 (*c*_*i*_ = *c*_*a*_−(*A*/*g*_*CO2*_) and *g*_*CO2*_ ≈ *g*_*s*_/1.6). This difference is indicative of greater photosynthetic capacity in FIL2. Fluorescence measurements indicated that FIL2 had a slightly higher Φ_PSII_, despite a lower fraction of PSII involved in photochemistry (*F*_v_′/*F*_m_′), a disparity linked with greater qP in FIL2 (Table 3). Since qP indicates turnover of electron carriers, which would be linked with demand for reducing equivalents by CO_2_ assimilation, this result further supports the indication of slightly greater photosynthetic capacity in FIL2. SPAD values indicated no significant difference in chlorophyll content between the parent lines (Table 3).

**Table 3:** Means (SE) leaf physiological trait values for FIL2, HAL2 and F_2_ *Panicum hallii* plants

Trait	*N*	FIL2	*N*	HAL2	LRT	*N*	F_2_	F_2_ range
SPAD	20	34.4 (0.64)	18	32.7 (0.69)	3	207	30 (0.44)	18.9–40.5
*A* (μmol CO_2_ m^−2^ s^−1^)	19	15.1 (2.35)	18	13.6 (2.23)	0.87	196	12.8 (2.00)	0.2–30.4
*g*_*s*_ (mol H_2_O m^−2^ s^−1^)	19	0.091 (0.0112)	18	0.102 (0.0112)	0.77	196	0.082 (0.0077)	0.019–0.184
c_i_ (μmol CO_2_ mol^−1^)	19	114 (17)	18	155 (17)	8.97**	196	127 (21)	17–281
qP	20	0.57 (0.042)	18	0.53 (0.042)	0.99	213	0.53 (0.041)	0.14–0.87
*F*_v_'/*F*_m_'	20	0.4 (0.0138)	18	0.417 (0.0141)	1.71	213	0.386 (0.0132)	0.26–0.55
Φ_PSII_	20	0.235 (0.024)	18	0.224 (0.0246)	0.3	213	0.207 (0.0226)	0.034–.408

*N,* number of replicates per parental lines or total number of F_2_ individuals (1 replicate per F_2_); LRT, Likelihood ratio test 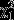, H_0_, no difference in parental means

** *P *<* *0.01. At ambient CO_2_: *A*, net CO_2_ assimilation rate; *g_s_*, stomatal conductance to water; *c*_*i*_, intercellular CO_2_ concentration; qP, photochemical quenching; *F*_v_'/F_m_', light adapted efficiency of energy harvesting by PSII; Φ_PSII_, quantum yield of PSII.

### Genetic map

**Fig 2:**
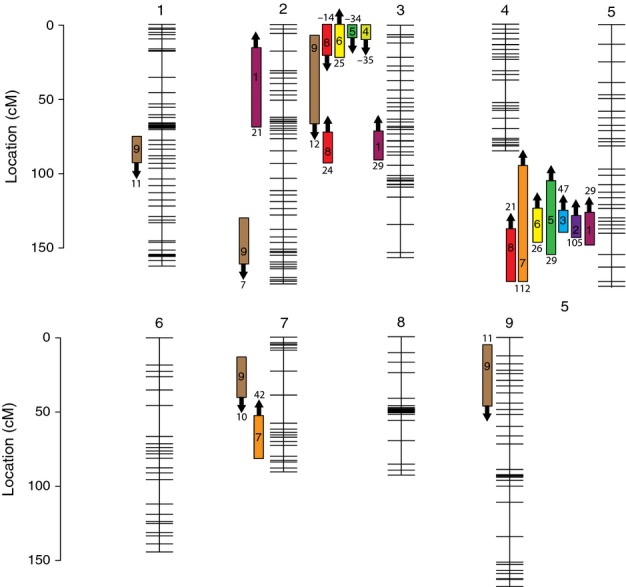
Genetic map of the FIL2 × HAL2 F_2_ hybrid population of *Panicum hallii* with the location of morphological trait quantitative trait loci (QTLs). Rectangular box indicates 1.5–LOD drop confidence intervals. Location of numbers within boxes is the location of QTL peaks. Arrow is the direction of additive effect, with an up arrow indicating that the FIL2 allele increases the trait value. Number above or below each QTL is the percentage of parental divergence (HAL2 vs FIL2) explained by the QTL. Traits: 1, tiller height; 2, number of tillers; 3, tiller width; 4, leaf length; 5, leaf width; 6, flowering time; 7, inflorescence length; 8, number of flowers; 9, seed mass. cM, centimorgans.

Our first round of linkage map construction in JoinMap utilized 782 out of the available 803 RAD-tag SNP markers. We recovered the nine linkage groups that were predicted from cytology (Gould, 1958; Waller, 1976). However, examination of this initial map revealed many marker order errors as well as poor markers with a high level of genotyping error. The genotyping errors appeared to be mostly the result of misidentification of heterozygote genotypes as homozygotes due to low sequence coverage at marker loci. After systematically purging poor markers using goodness of fit criteria (mean chi-squared contribution > 3) and close examination of recombination fraction plots, 278 remained for the second round of map construction. This linkage map had a combined length of 1252.1 cM and utilized recombination events from 257 F2 hybrids (Figs 2, S5). There was segregation distortion across many of the remaining markers, with 156 (56%) markers distorted at *P *<* *0.05, 110 (40%) at *P *<* *0.01, 66 (24%) at *P *<* *0.001, and 36 (13%) at *P *<* *0.0001.

**Fig 3:**
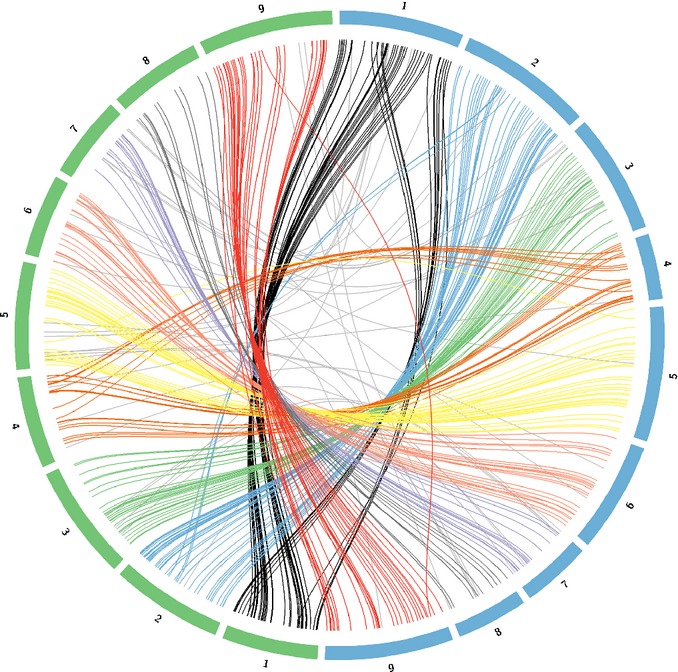
Synteny plot of marker positions from *Panicum hallii* genetic map (blue) with predicted homologous positions in the foxtail millet (*Setaria italica*) genome (green) suggests a high level of conserved synteny between the two species. Chromosome numbers are labeled 1–9 for both species.

**Fig 4:**
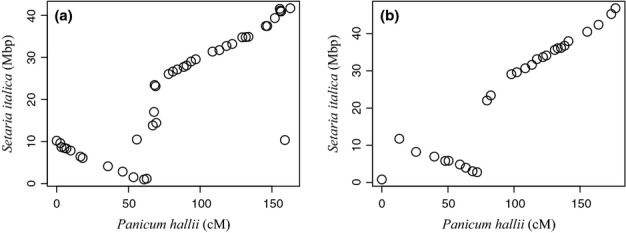
Comparisons of marker positions from *Panicum hallii* genetic map with predicted homologous positions in the foxtail millet (*Setaria italica*) genome (physical map) revealed large inversions between the two species on chromosomes (a) 1 and (b) 5. Mbps, mega base pairs; cM, centimorgans.

Comparisons of the marker location between *P. hallii* and their predicted location in the foxtail millet genome revealed a high level of conserved synteny with no clear evidence for translocations between chromosomes (Fig.3). However, we did observe two large-scale chromosomal inversions between the species on linkage groups 1 and 5 in dot plot comparisons between the physical map location of the foxtail millet genome and the linkage map location for *P. hallii* (Fig.4).

### QTL analyses

Overall, we identified 28 QTLs from 14 phenotypic traits (nine morphological traits and five physiological traits) and hybrid sterility in the best-fit stepwise QTL models (Fig.2, Table 4). The vast majority of morphological QTLs (85%) had additive effects in the same direction as the parental divergence. See Tables S2 and S3 for R/qtl input files for QTL mapping.

**Table 4:** Quantitative trait loci (QTLs) and their main effects from the best-fit models of stepwise analyses for the FIL2 × HAL2 F_2_ population of *Panicum hallii*

Trait	Chr	Peak	1.5–LOD interval	LOD	a	D	%var	%div
Tiller height	2	38	20–71	5.387	3.172	−0.4	6.695	21.083
Tiller height	3	77	73–85	9.361	4.338	−2.045	12.096	28.833
Tiller height	5	140	124–148	10.731	4.43	0.436	14.057	29.445
Number of tillers	5	137	126–143	4.359	2.037	−0.055	8.024	105.000
Tiller width	5	127	123–139	8.386	0.163	0.025	14.863	47.246
Leaf length	3	6	0–11	4.025	−1.753	−0.361	7.463	−35.201
Leaf width	3	6	0–10	5.51	−0.491	−0.172	9.272	−33.516
Leaf width	5	129	105–154	4.915	0.43	−0.28	8.223	29.352
Flowering time	3	15	1–32	5.15	5.15	−3.013	6.4	24.789
Flowering time	5	132	119–146	9.901	5.413	−1.838	7.437	26.055
Inflorescence length	5	161	93–169	5.165	1.582	0.682	9.013	111.802
Inflorescence length	7	66	55–81	4.199	0.599	1.203	7.256	42.332
Number of flowers	3	18	0–27	3.912	−29.391	−6.714	5.748	−14.294
Number of flowers	3	80	75–91	8.919	48.727	−10.939	13.787	23.698
Number of flowers	5	148	136–169	6.059	42.351	−1.959	9.098	20.596
Seed mass	1	83	75–90	5.506	−0.046	0.014	8.629	11.220
Seed mass	2	152	131–161	3.989	−0.03	0.035	6.139	7.317
Seed mass	3	14	9–68.907	5.155	−0.049	0.008	8.045	11.951
Seed mass	7	26	15–39	4.34	−0.039	−0.012	6.707	9.512
Seed mass	9	19	9–47	5.633	−0.047	0.008	8.842	11.463
Sterility	2	65	64–71	20.06	−0.1	0	27.452	na
Sterility	7	29	16–39	4.89	−0.101	0	5.708	na
Sterility	7	83	81–84	25.352	−0.107	0	36.737	na
SPAD	3	1	0–13	4.073	−1.425	−1.339	8.724	na
*A* (μmol CO_2_ m^−2^ s^−1^)	3	94.4	89–107	3.844	2.818	−1.528	8.808	na
*g*_*s*_ (mol H_2_O m^−2^ s^−1^)	3	94.4	90–106	3.969	0.014	−0.009	9.076	na
Φ_PSII_	3	94.4	88–107	4.54	0.035	−0.017	9.564	na
qP	3	95	89–141	4.30	0.068	−0.025	9.157	na

Chr, linkage group; Peak, location of the QTL peak; LOD, logarithm of odds; a, additive effect; D, dominance deviation; %var, percent of variance explained; %div, percent of parental divergence explained (only calculated for traits that showed significant differences between FIL2 and HAL2); *A*, net CO_2_ assimilation rate; *g_s_*, stomatal conductance to water; Φ_PSII_, quantum yield of PSII; qP, photochemical quenching; na, not applicable.

A large proportion (79%) of QTLs identified in our study had overlapping 1.5 LOD-drop confidence intervals with at least one other QTL. This is a high level of clustering, given that only 31% of the genome was occupied by the 1.5 LOD-drop of any QTL. Five morphological traits had overlapping confidence intervals on one region of linkage group 3, while seven morphological traits had overlapping confidence intervals on linkage group 5. Similarly, we mapped colocalizing physiological QTLs to a region of linkage group 3 for four physiological traits: *A,* gs, Φ_PSII_ and qP (Table 4).

We mapped colocalizing QTLs for five traits to one region of linkage group 5 (Fig.2, Table 4). QTLs for infloresence length and flower number also had an overlapping confidence interval in this region, but the peak of these QTLs was offset from the other five. All five of the colocalizing QTLs had major phenotypic effects, explaining over 15% of the phenotypic divergence between the two ecotypes. This locus, or linked set of loci, accounted for 47% of the divergence between HAL2 and FIL2 in tiller width, 29% of the divergence in both tiller height and leaf width, 26% of the divergence in flowering time and 105% of the divergence in number of tiller. All seven of the QTLs that mapped to this region of linkage group 5 had additive effects in the same direction as the phenotypic divergence of the ecotypes, where the FIL2 allele made each trait larger.

The clustering of morphological QTLs on linkage group 3 was different from that of linkage group 5, as only QTLs for seed size and flowering time were in the same direction as the ecotype divergence. The three other QTLs in this region, for leaf length, leaf width and number of flowers, had additive effects in the opposite direction of the ecotype divergence. In other words, the FIL2 allele contributed to smaller trait values for these three traits.

Only six morphological QTLs did not have overlapping confidence intervals with other morphological QTLs (Fig.2). Of these six independent QTLs, four of them contributed to seed size. Further, all five of the seed size QTLs had moderate effects, each explaining only 7–11% of the parental divergence in this trait. Further, the seed size QTLs all had additive effects in the same direction, with the FIL2 allele contributing to smaller seeds at all loci. Finally, it should be noted that seed size did not have a QTL in the vicinity of the large effect colocalizing QTLs on linkage group 5.

Synteny mapping demonstrated that the clustering of large effect QTLs on linkage group 5 in *P. hallii* colocalized with a flowering time QTL discovered in a recent study of foxtail millet (Mauro-Herrera *et al.*, 2013). The foxtail millet study identified a number of flowering time candidate genes in this region of synteny, including homologues of FVE/OsFVE (Ausin *et al.*, 2004; Baek *et al.*, 2008), OsLFL1 (Peng *et al.*, 2007, 2008), SPA1 (Laubinger *et al.*, 2006), and OsFTL9/ZCN12 (Danilevskaya *et al.*, 2008). The linkage group 5 QTL of *P. hallii* is also in the vicinity of a homologue of developmental gene *MORE AXILLARY BRANCHES1* (*MAX1*).

**Fig 5:**
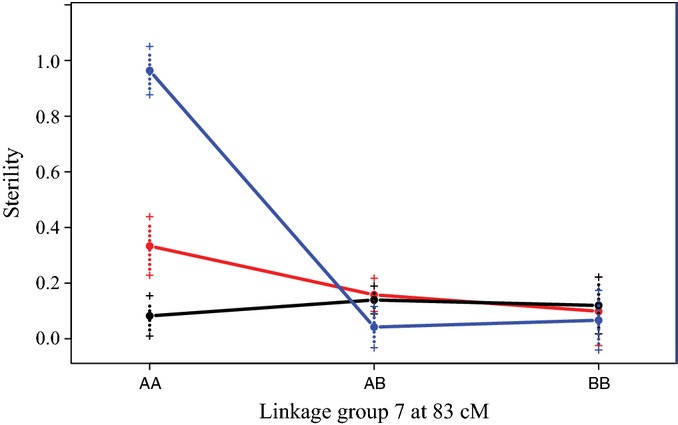
A two-locus Dobzhansky–Muller hybrid sterility incompatibility system between the HAL2 and FIL2 lines of *Panicum hallii*. Mean genotypic effects for the homozygous HAL2 individuals (AA), homozygous FIL2 individuals (BB), and heterozygous individuals (AB) are indicated for the locus on linkage group 7 on the *x*-axis and by colored lines (AA, red line; BB, blue line; AB, black line) for the locus on linkage group 2 at 65 cM. A high level of sterility occurs in individuals that are homozygous for the HAL2 allele on linkage group 7 and homozygous for the FIL2 allele on linkage group 2. cM, centimorgan.

Stepwise QTL model fitting revealed a significant (LOD = 17.274) pairwise epistatic interaction for hybrid sterility established by seed set (Fig.5). This pairwise interaction appears to be a classic Dobzhansky–Muller incompatibility (Coyne & Orr, 2004; Sweigart & Willis, 2012; Bomblies, 2013). Here, individual plants were sterile when homozygous for the HAL2 allele at position 83 cM on linkage group 7 and homozygous for the FIL2 allele at position 65 cM on linkage group 2.

## Discussion

Determining the genetic architecture of traits involved in the adaptive divergence between ecotypes is crucial to understanding the process of speciation. In this study, we used genetic mapping to evaluate whether or not QTLs for traits involved in the divergence between xeric and mesic ecotypes of *P. hallii* colocalize to particular genomic regions. In the process of conducting this study, we assembled the first genetic map for *P. hallii*, which revealed a high level of synteny with foxtail millet. We mapped at least one QTL for every morphological trait, as well as QTL for five out of seven physiological traits. We found large effect colocalizing QTLs on linkage group 5, which controlled multiple classic ecotype-differentiating traits including tiller and leaf size and shape as well as flowering time. We also identified a two-locus epistatic hybrid incompatibility system that causes sterility in hybrids. Overall, our study suggests that some ecotype-differentiating traits evolve independently while others may evolve in tandem due to genetic colocalization resulting from linkage or pleiotropy.

### The genetics of divergence between ecotypes

The evolution of plant ecotypes adapted to xeric vs mesic habitats is a common phenomenon that serves as a model of how ecology can contribute to the process of speciation (Clausen, 1951; Clausen & Heisey, 1958; Grant, 1981; Kruckeberg, 1986; Rajakaruna, 2004; Lowry, 2012). One of the key discoveries of our study was the colocalization of a major QTL for five traits to the same region of linkage group 5. The effects of this region on multiple traits could be due to tight linkage of multiple genes with effects on individual traits, or could be caused by pleiotropic effects of a single gene on multiple traits. Regardless, it is interesting that so much of the phenotypic divergence between the ecotypes maps to one genomic location and that the additive effects of these QTLs are all in the same direction as the ecotype trait divergences. The linkage group 5 QTLs contrasts with those that clustered on linkage group 3, where the additive effects of three of the QTLs were in the opposite direction of the ecotype divergence.

The finding that multiple major-effect ecotype-differentiating QTLs colocalize to linkage group 5 suggests that the evolution of these traits is nonindependent. This could be the result of fundamental trade-offs between above ground growth and the demand for water imposed by transpiration (Geber & Dawson, 1990; Dudley, 1996; Ackerly *et al.*, 2000). There may be structural developmental reasons for those trade-offs, especially if the same genetic loci are contributing to evolution of many of these traits. One possible reason why multiple ecotype-differentiating traits have colocalizing QTLs could be that those traits can be simultaneously altered by mutations in major developmental genes and hormone pathways. In fact, mutations in hormone pathways result in phenotypic effects that mimic the developmental divergence between xeric and mesic ecotypes (Fleet & Sun, 2005; Domagalska *et al.*, 2007; Zhao, 2010). Further, recent studies in rice (Xue *et al.*, 2008; Yan *et al.*, 2011) have cloned the QTLs that pleiotropically control flowering time, plant height, and the number of seeds per panicle in the same additive directions as the colocalizing QTLs on linkage group 5 in *P. hallii*. Thus, there is precedent that the set of traits involved in xeric/mesic ecotype divergence can be controlled pleiotropically in other grass species. We identified multiple potential candidate genes underlying the pleiotropic QTL on linkage group five through synteny analysis between *P. hallii* and foxtail millet. The gene *MAX1*, which is found in this region, is known to repress axillary bud primordia and control branching in *A. thaliana* (Stirnberg *et al.*, 2002; Waters *et al.*, 2012) and is thus a candidate gene underlying the QTLs that colocalize in this region.

Another possible reason why the QTLs for so many traits colocalize to the same genomic region could be that haplotypes of multiple alleles are held together by a chromosomal inversion (Kirkpatrick, 2010; Lowry & Willis, 2010). A set of ecotype-differentiating traits, similar to those found in *P. hallii*, all mapped to a single chromosomal inversion polymorphism in *Mimulus guttatus* (Hall *et al.*, 2006, 2010; Lowry & Willis, 2010). While we found no evidence that a chromosomal rearrangement is in the vicinity of the colocalizing QTLs on linkage group 5, we cannot yet rule out this possibility without further fine genetic mapping.

Four of the five QTLs for seed size did not have overlapping confidence intervals with any other QTLs. Thus, this trait may be able to evolve more independently of many of the other ecotype-differentiating morphological traits, especially those that colocalize on linkage group 5. Classic studies have identified associations between seed size and many environmental factors (Baker, 1972; Salisbury, 1974). Baker (1972) surveyed seed size in nearly 2500 plant taxa in California and found plants typically had larger seed sizes in locations where there was a greater likelihood of exposure to drought after germination. Provisioning in larger seeds can provide a seedling with greater potential to establish a large root system before the onset of periods of low water availability (Kneebone & Cremer, 1955; Glewen & Vogel, 1984; Venable & Brown, 1988). *P. hallii* follows this classic pattern with larger seeds in the xeric var. *hallii* and smaller seeds in the mesic var. *filipes*. Our study suggests a highly polygenic genetic architecture involved in seed size evolution in this system, with the five significant moderate effect QTLs only accounting for *c*. 51% of the divergence between the two varieties. This finding may also be relevant to the closely related bioenergy crop *Panicum virgatum* (switchgrass), where seed size is a focal trait for breeding because it is positively correlated with seedling establishment (Aiken & Springer, 1995; Smart & Moser, 1999; Boe, 2003). Just like *P. hallii, P. virgatum* has a wet lowland habitat ecotype with smaller seeds than its dry habitat upland ecotype (Seepaul, 2010; Lowry *et al.*, 2014).

### Genetics and physiology

Regulation of photosynthesis and stomatal gas exchange is crucial for plant response to drought (Hiesey & Milner, 1965; Ackerly *et al.*, 2000; Chaves *et al.*, 2009; Pinheiro & Chaves, 2011; Taylor *et al.*, 2011, 2014). We found that FIL2 showed greater capacity to draw down CO_2_ concentrations inside the leaf, supported by consistent but nonsignificant differences in chlorophyll fluorescence traits, and indicating greater intrinsic water-use efficiency than HAL2. Given the greater canopy size of FIL2 individuals, and the expectation that transpiration will scale with the canopy size, lower *g*_*s*_ may help to reduce the possibility of hydraulic failure in FIL2 (Taylor *et al.*, 2014). Equally, as HAL2 flowers significantly more quickly than FIL2, greater *g*_*s*_ in HAL2 may help to improve the return on investments in shorter-lived leaves (Wright *et al.*, 2003).

Although we were able to identify a colocalizing QTL for four physiologically inter-linked traits, we do not yet know how the two varieties respond to decreased levels of available soil moisture, or have data on leaf lifespan and structural investment, which will be crucial in understanding the evolution of divergence between *P. hallii* ecotypes. The morphological and physiological traits underlying photosynthetic performance and water-use efficiency are notoriously plastic (Flood *et al.*, 2011). Our physiological measurements were made on flowering plants in a glasshouse under well-watered conditions, but genetic differences contributing to divergence between *P. hallii* ecotypes may also manifest at particular ontogenetic stages and/or as plastic responses to decreasing soil water availability (Teng *et al.*, 2004; Van Kleunen & Fischer, 2005; Picotte *et al.*, 2007; Maherali *et al.*, 2009; Pinheiro & Chaves, 2011).

One of the most striking results of our study was that while most of the physiological traits did not strongly differ between HAL2 and FIL2, many of these traits were highly transgressive in their segregation in the F_2_ hybrids. Transgressive segregation is often caused by alleles with opposite additive effects across loci being shuffled into different combinations through hybridization (Rieseberg *et al.*, 1999; Lexer *et al.*, 2005). Morphological traits were less transgressive than the physiological traits. Even so, we did identify morphological QTLs (leaf width and number of flowers) that had opposite additive effects consistent with transgressive segregation.

### Reproductive isolation between the ecotypes

Understanding what reproductive isolating barriers are important for reducing gene flow between ecotypes is key to better understanding the process of speciation (Coyne & Orr, 2004; Nosil *et al.*, 2005; Sobel *et al.*, 2010). *P. hallii* var. *hallii* and var. *filipes* have an overlapping geographic range in southern Texas and northern Mexico and occur in sympatry in areas where both dry upland and wet lowland habitats exist in close proximity (Waller, 1976). We have now identified multiple potential reproductive isolating barriers that could explain the maintenance of the ecotypes in sympatry.

Approximately one-fifth of the F_2_ hybrids had a high level of sterility. This hybrid sterility is partially caused by a two-locus nuclear Dobzhansky–Muller incompatibility between linkage groups 2 and 7. Dobzhansky–Muller incompatibilities are thought to play a major role in the evolution of new species by causing postzygotic reproductive isolation (Coyne & Orr, 2004) and are frequently found in genetic mapping studies between diverging plant varieties and species (Moyle & Graham, 2005; Sweigart *et al.*, 2006; Mizuta *et al.*, 2010; Kubo *et al.*, 2011). In some cases, the genes underlying these epistatic incompatibility systems in plants have even been identified (reviewed in Rieseberg & Blackman, 2010; Sweigart & Willis, 2012; Bomblies, 2013). The incompatibility system we identified here required homozygote genotypes for both varieties at each of two loci. The relatively low level of hybrid sterility, and no evidence of hybrid inviability, suggests that postzygotic isolation may not be the main factor maintaining the distinctness of the *P. hallii* varieties.

Ecological isolating barriers often form as a byproduct of divergent adaptations between ecotypes and species (Coyne & Orr, 2004; Nosil, 2012). The differences in flowering time between the ecotypes suggest that temporal isolation may be a significant barrier to gene flow. Further, the fact that var. *hallii* and var. *filipes* are found in different microhabitats suggests a potential role for habitat isolation and extrinsic postzygotic isolation as barriers to gene flow. Extrinsic postzygotic isolation can also result from transgressive segregation, where increased phenotypic variability leads to the production of maladapted hybrids (Rogers & Bernatchez, 2006; Renaut *et al.*, 2009). Hybrids between the two ecotypes were highly transgressive in physiological traits, which could lead to decreased fitness in nature.

The most important barrier for *P. hallii* may be mating system isolation. Our recent population genetic survey of *P. hallii* revealed that it has a near obligate self-fertilization mating system (Lowry *et al.*, 2013). We have yet to identify a population of *P. hallii* with an appreciable level of outcrossing. The low level of polymorphism in the genome sequence of the FIL2 accession produced for this study further supports the idea that *P. hallii* plants typically occur in nature as highly inbred nearly-homozygous lines as a result of their selfing mating system. With such low levels of outcrossing, the possibility of hybridization between the varieties is very low. Martin & Willis (2007) recently showed that mating system can be a very strong barrier to gene flow, even when only one out of a pair of hybridizing species has a high rate of self-fertilization.

### Conclusions

It has long been known that a common set of traits often differentiates xeric and mesic ecotypes across plant species (Clausen & Heisey, 1958). Our study reinforces the pattern found recently in other systems (Latta & Gardner, 2009; Lowry & Willis, 2010; Lovell *et al.*, 2013) that QTLs for ecotype-differentiating traits frequently colocalize to the same genomic position. The same set of ecotype-differentiating traits found between xeric and mesic varieties of *P. hallii* are also involved in the divergence of upland and lowland ecotypes of the closely related bioenergy crop switchgrass (*P. virgatum*; Lowry *et al.*, 2014). Gaining insight into the molecular details of colocalizing QTL in these systems will be an important next step in understanding the factors constraining or facilitating adaptation and ecotypic differentiation to different habitats in nature.

## Supporting Information

Fig S1 Flowchart of the pipeline for mapping and SNP calling of RAD-tag markers for the FIL2 × HAL2 hybrid F2 mapping population of *Panicum hallii*. **Fig. S2** Flowchart of the pipeline for synteny analysis between the *Panicum hallii* linkage map and the genome of foxtail millet (*Setaria italica*). **Fig. S3** Histograms of phenotypic variation for morphological traits in the F2 hybrid population of *Panicum hallii*. **Fig. S4** Histograms of phenotypic variation for physiological traits in the F2 hybrid population of *Panicum hallii*. **Fig. S5** Plot of recombination fractions across the *Panicum hallii* FIL2 × HAL2 genetic map. **Methods S1** Supplementary methods.

Table S1 Summary of Illumina libraries used in the preliminary Meraculous *Panicum hallii* assembly **Table S2** R/qtl input file for QTL mapping of morphological traits **Table S3** R/qtl input file for QTL mapping of physiological traits
