# Supplementary material for: The genetics of divergence and reproductive isolation between ecotypes of Panicum hallii
Source: New Phytol. 2014 Sep 23;205(1):402–14. doi: 10.1111/nph.13027 (PMC4265272; doi:10.1111/nph.13027)
Supplement: Fig S1 — Flowchart of the pipeline for mapping and SNP calling of RAD-tag markers for the FIL2 × HAL2 hybrid F2 mapping population of Panicum hallii. Fig. S2 Flowchart of the pipeline for synteny analysis between the Panicum hallii linkage map and the genome of foxtail millet (Setaria italica). Fig. S3 Histograms of phenotypic variation for morphological traits in the F2 hybrid population of Panicum hallii. Fig. S4 Histograms of phenotypic variation for physiological traits in the F2 hybrid population of Panicum hallii. Fig. S5 Plot of recombination fractions across the Panicum hallii FIL2 × HAL2 genetic map. Methods S1 Supplementary methods. [file nph0205-0402-SD1.docx]

**Supporting Information Figs S1–S5 and Methods S1**

**Fig. S1** Flowchart of the pipeline for mapping and SNP calling of RAD-tag markers for the FIL2 × HAL2 hybrid F_2_ mapping population of *Panicum hallii*.

**
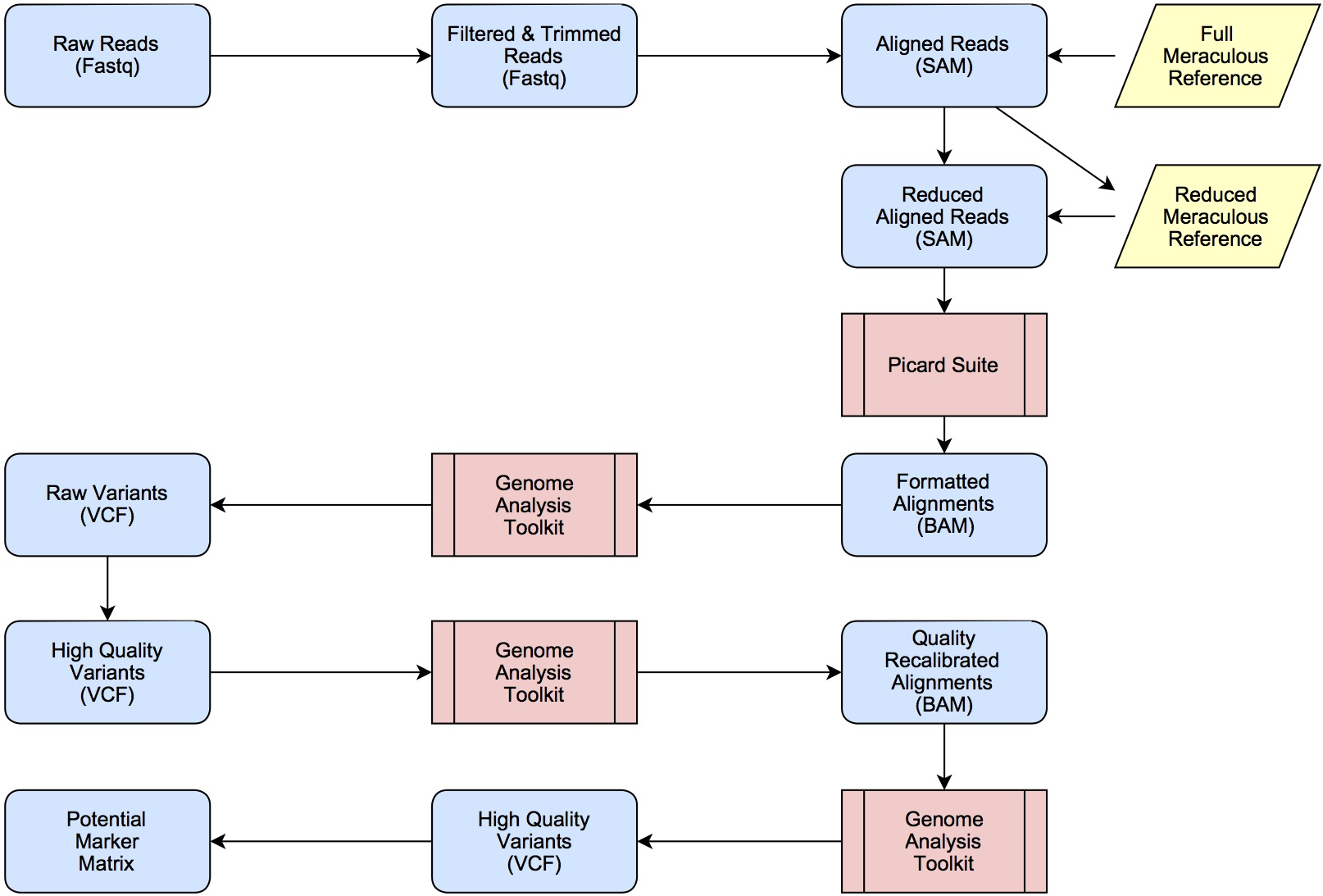
**

**Fig. S2** Flowchart of the pipeline for synteny analysis between the *Panicum hallii* linkage map and the genome of foxtail millet (*Setaria italica*).

**
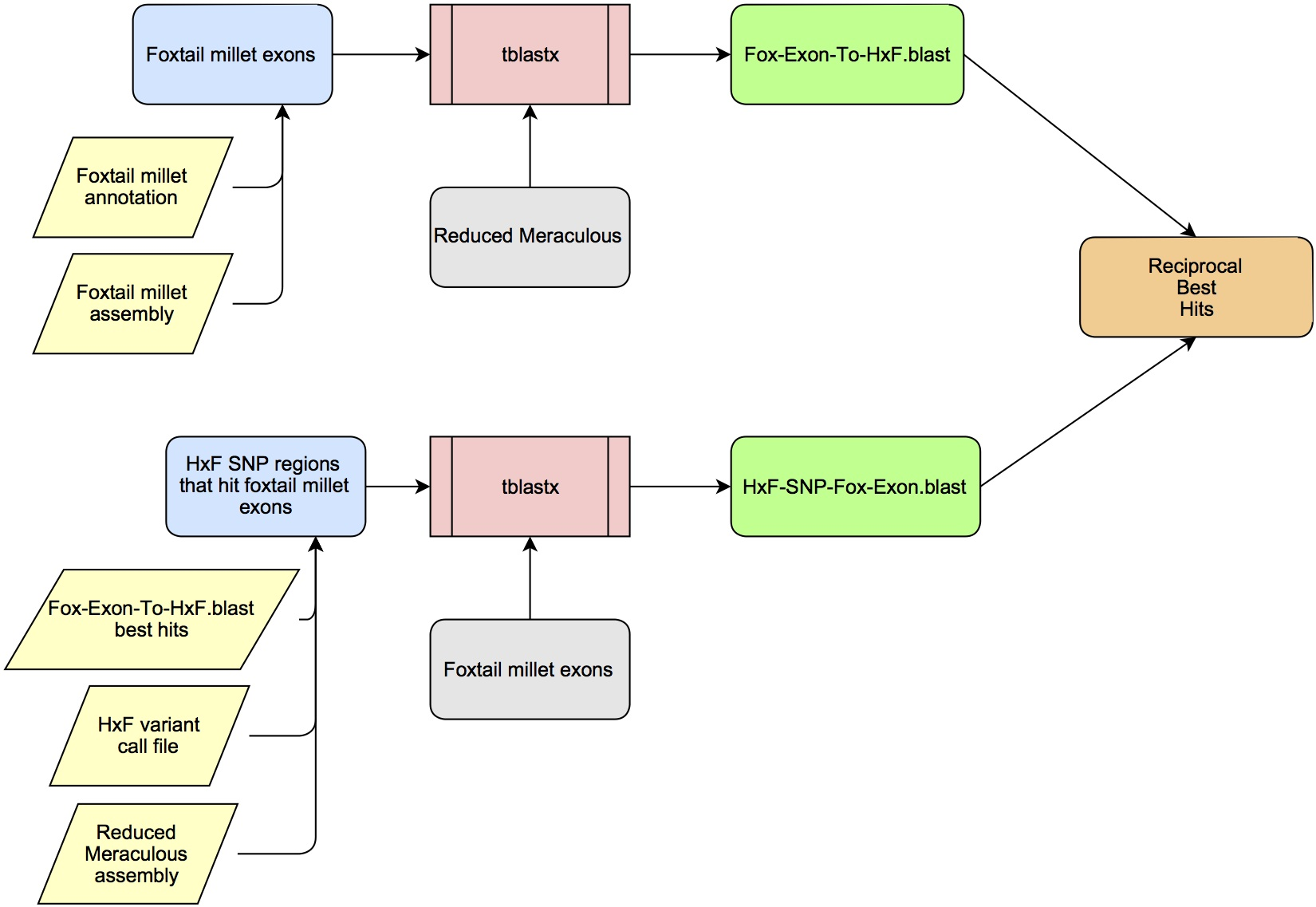
**

**Fig. S3** Histograms of phenotypic variation for morphological traits in the F_2_ hybrid population of *Panicum hallii*. Mean values for the HAL2 (circle) and FIL2 (square) parents are shown.

**
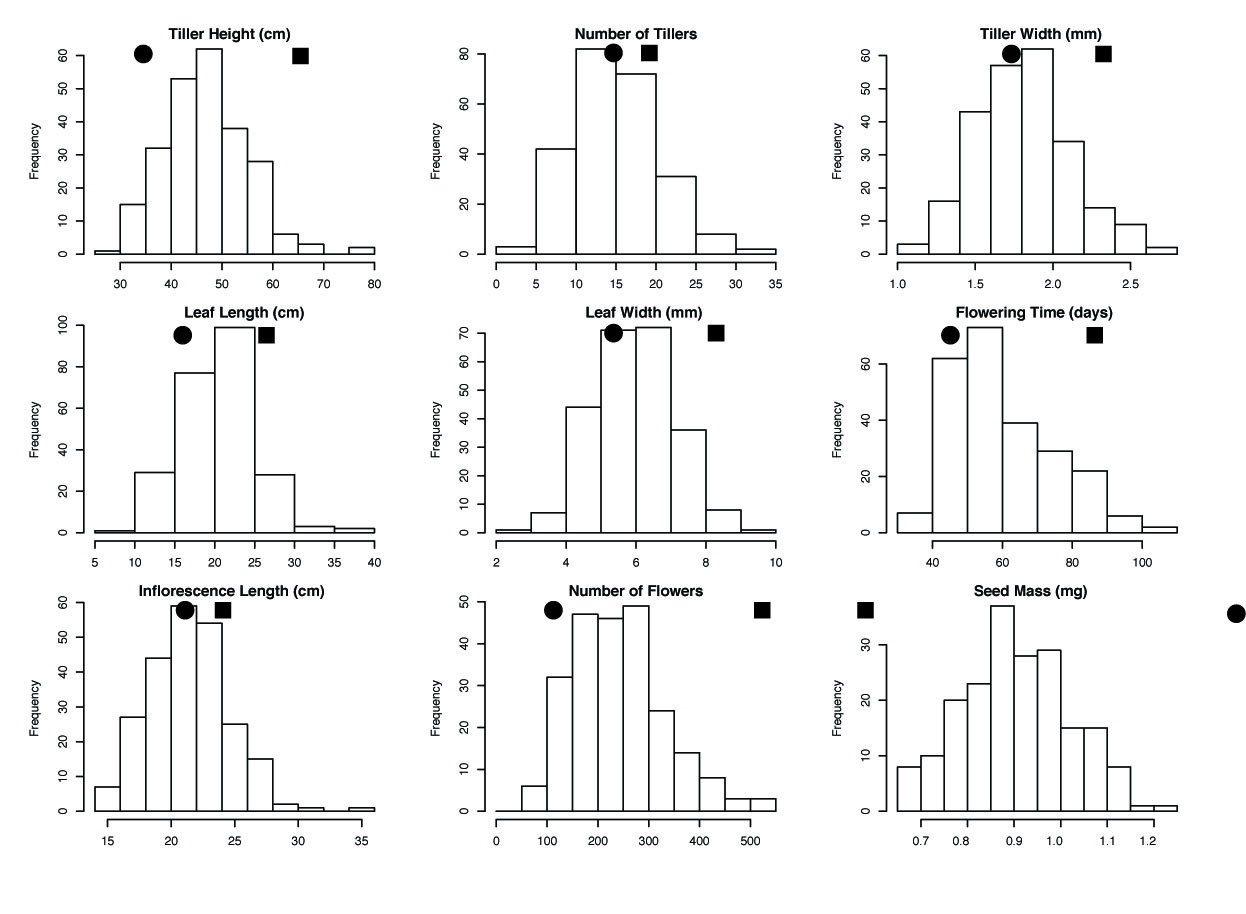
**

**Fig. S4** Histograms of phenotypic variation for physiological traits in the F_2_ hybrid population of *Panicum hallii*. Mean values for the HAL2 (circle) and FIL2 (square) parents are shown.

**
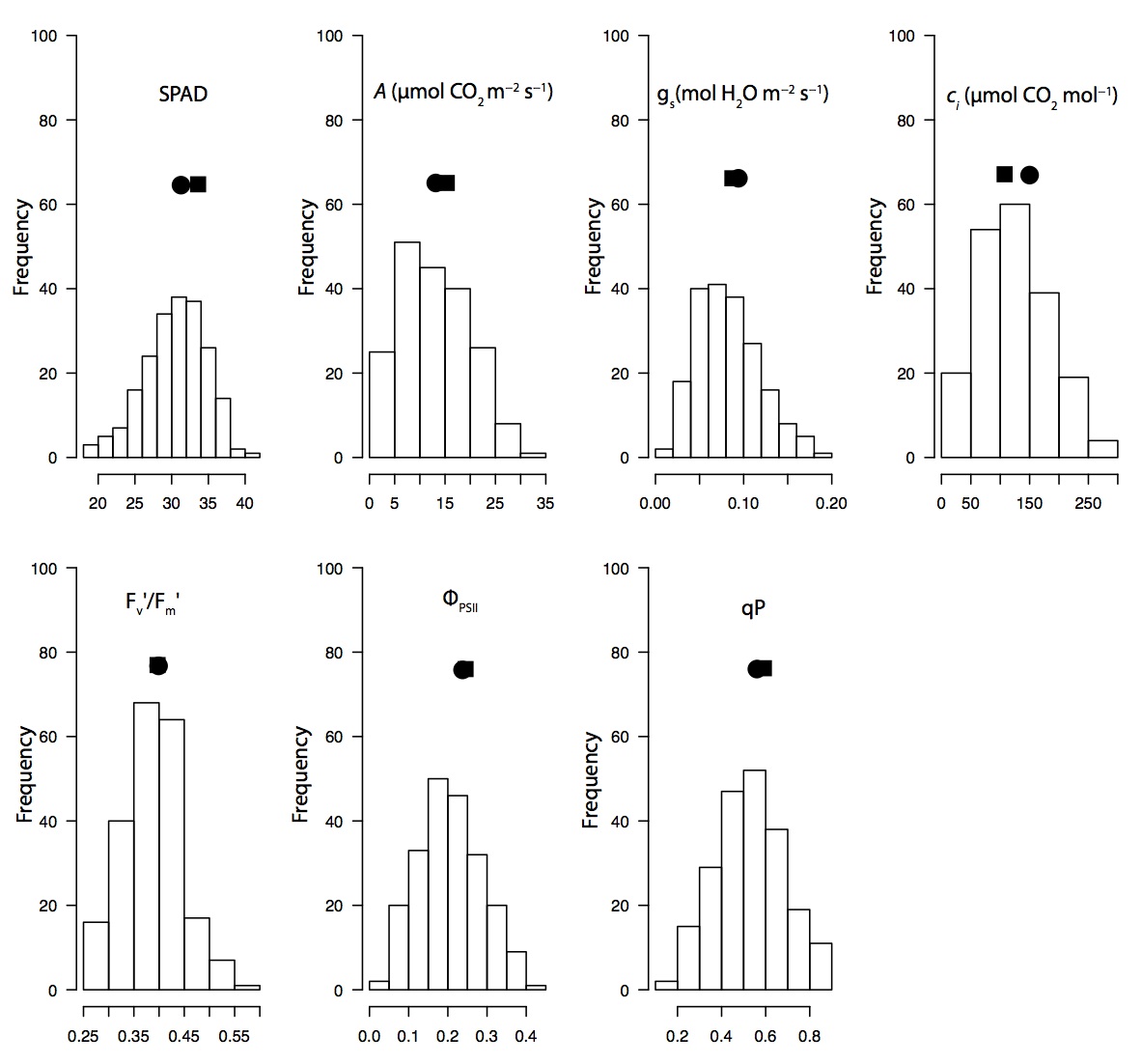
**

**Fig. S5** Plot of recombination fractions across the *Panicum hallii* FIL2 × HAL2 genetic map.

**
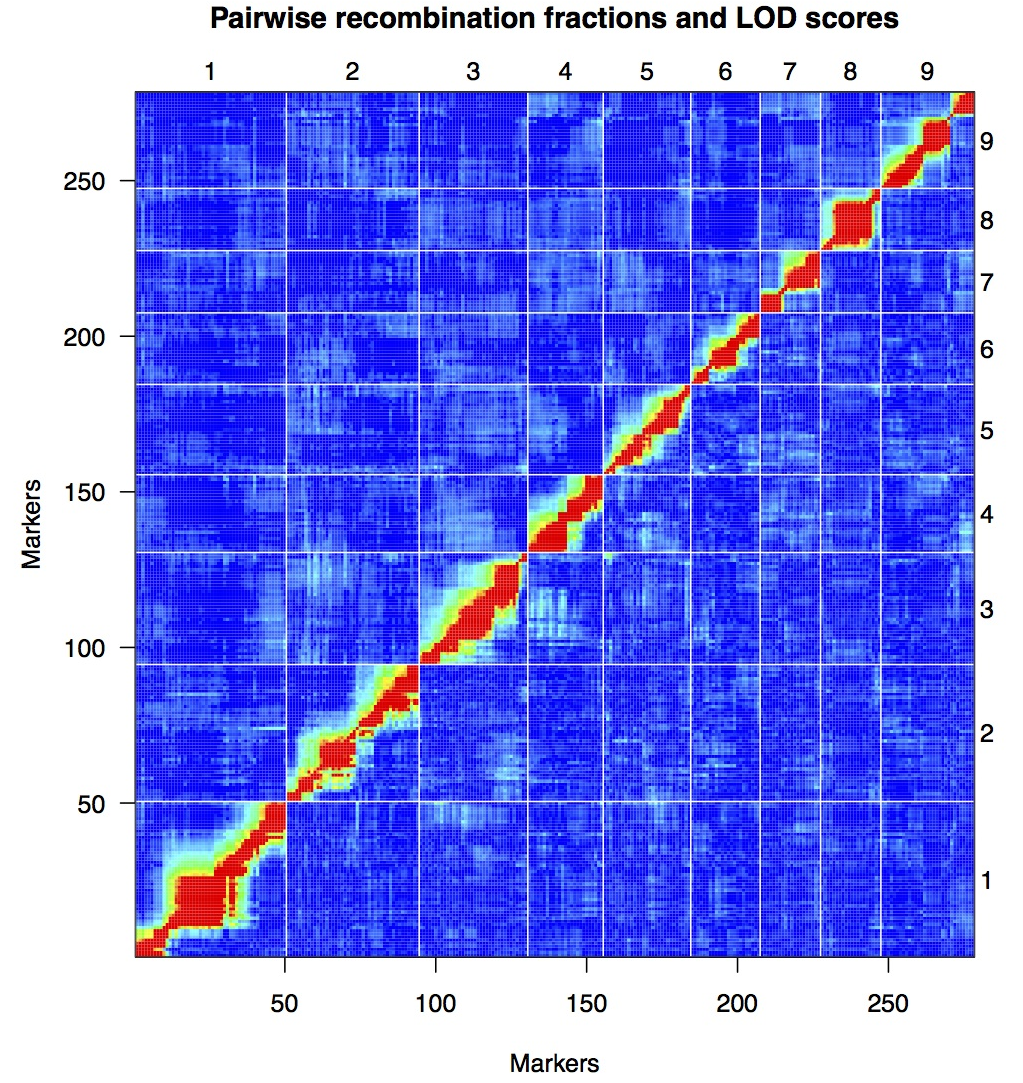
**

**Methods S1**

**Crossing methodology**

Since *P. hallii* is a near obligate self-fertilizing species (Lowry *et al*., 2012, 2013) it was necessary to develop an emasculation method to conduct crosses between the varieties. The day before crossing, all previously opened flowers were removed. Plants were placed in the dark overnight to synchronize flower opening, and were removed from dark the following morning between 08:00–10:00 h. We carefully separated the lemma and palea of opening florets under a microscope with sterile fine forceps, which resulted in the release of the anthers and stigma. Anthers were quickly removed with forceps before anther dehiscence and pollen release. The emasculated flower was marked and set aside for pollination before fresh pollen was collected from the sire of the cross. The HAL2 plant was the dam in the cross to FIL2 because the flowers of *P. filipes* are difficult to emasculate given their small size (Lowry *et al*., 2013). *Panicum* pollen is known to have a very short life span (half-life for related *P. virgatum*: 9.3–40.4 min depending on temperature; Ge *et al*., 2011), so we completed crosses as quickly as possible. Pollinated flowers were marked and monitored daily until seed maturation. Putative F_1_ plants derived from these seeds were grown in the University of Texas Welch glasshouses and eight microsatellite loci were genotyped (Lowry *et al*., 2012) to confirm hybrids. We obtained a single F_1_ hybrid between HAL2 and FIL2 and collected open-pollinated seeds from the F_1_ plant for the F_2_ generation.

**Physiology measurement conditions**

After morphological phenotyping, the F_2_ plants were transplanted to 3.7 l pots in December 2011. The plants were moved outdoors at the University of Texas Brackenridge Field Laboratory in March 2012, and periodically repotted to maintain vigorous root growth. To provide for consistent and controlled conditions during gas exchange measurements, plants were moved into the adjacent University of Texas Brackenridge Field Laboratory glasshouses on 29 June 2012.

Prior to physiological measurements, plants were watered daily and measurements were made on 10 July–19 July. Supplemental lighting and glasshouse climate controls resulted in (mean ± SD) daily integrated Photosynthetic Photon Flux Density (PPFD) of 14 ± 3.1 mol m^−2^ d^−1^, daily maximum PPFD of 1149 ± 121 µmol m^−2^ s^−1^, daily mean temperature of 28.0 ± 1.33°C, and daily mean relative humidity of (RH) 78 ± 4.8%.

Measurements of photosynthesis were made using two LI-6400XT portable photosynthesis systems equipped with leaf chamber fluorometers (LI-6400-40, LI-COR Inc, Lincoln NE, USA). Greenhouse conditions during measurement periods (10:30 h until 15:30 h daily) were (mean ± SD) PPFD 1110 ± 181 μmol m^−2^ s^−1^, mean temperature 30.2 ± 0.99°C, and mean RH 68 ± 6%. Two or three flag leaves of flowering tillers from each plant were measured together in the cuvette to ensure that the entire surface area was filled with leaf material. Cuvette temperature was set to 30°C. The concentration of water vapor in the leaf chamber was controlled manually, to obtain values between 27–30 mol mol^−1^, resulting in leaf vapor pressure deficits (VPD) of 2.0 ± 0.21 kPa (mean ±SD). Preliminary light response curves, measured on six individuals, indicated an appropriate saturating level of PPFD to be 1000 μmol m^−2^ s^−1^.

**References**

**Ge Y, Fu C, Bhandari H, Bouton J, Brummer EC, Wang ZY. 2011.** Pollen viability and longevity of switchgrass (*Panicum virgatum* L.). *Crop Sci.* **51:** 2698–2705.

**Lowry DB, Purmal CT, Juenger TE. 2013.** A population genetic transect of *Panicum hallii* (Poaceae). *Am. J. Bot.* **100:** 592–601.

**Lowry DB, Purmal CT, Meyer E, Juenger TE. 2012.** Microsatellite markers for the native Texas perennial grass, *Panicum hallii* (Poaceae). *Am. J. Bot.* **99:** e114–e116.
